# The planning of a national breastfeeding educational intervention for medical residents

**DOI:** 10.3402/meo.v20.26380

**Published:** 2015-02-04

**Authors:** Catherine M. Pound, Katherine A. Moreau, Francine Hart, Natalie Ward, Amy C. Plint

**Affiliations:** 1 Children’s Hospital of Eastern Ontario, Ottawa, ON, Canada; 2 Children’s Hospital of Eastern Ontario Research Institute, Ottawa, ON, Canada

**Keywords:** educational intervention, postgraduate medical education, breastfeeding

## Abstract

**Background:**

Breastfeeding is the ideal form of nutrition for newborns, yet our recent pan-Canadian study showed that the knowledge, attitudes, and beliefs of primary care pediatricians and family physicians are suboptimal with regard to breastfeeding.

**Objective:**

We aim to develop, implement, and evaluate a national breastfeeding educational intervention at the postgraduate residency level.

**Methods:**

Our initial development process is informed by Kern’s approach to curriculum development. To date, we have completed breastfeeding education needs assessment surveys of both practicing physicians and medical residents. We have also developed learning outcomes as well as possible strategies for implementing and evaluating this future educational intervention.

**Results:**

The results of our needs assessment surveys provided a rationale to develop a breastfeeding educational intervention for medical residents. Through stakeholder consultations, we have developed five initial learning outcomes for a national breastfeeding educational intervention. We have also identified promising strategies for implementing and evaluating the intervention.

**Conclusions:**

This systematic process has provided an opportunity to create a national breastfeeding educational intervention for medical residents. It has fostered collaboration between experts and knowledge users, with the goal of impacting breastfeeding rates and duration of women, which will lead to improved maternal and child outcomes.

Breastfeeding is the ideal form of nutrition for newborns, and exclusive breastfeeding should be protected, promoted, and supported for the first 6 months after birth (1–3). Breastfeeding mothers rely on their physicians for support and advice, and studies show that recommendations of physicians and attitudes toward breastfeeding directly impact the success of a woman in breastfeeding (4–7). Yet, physicians and postgraduate trainees worldwide lack the skills and knowledge to properly guide and support lactating mothers (8–11). In Canada, primary care pediatricians and family physicians were recently found to have suboptimal beliefs, attitudes, and knowledge with regards to breastfeeding, with most physicians stating that their postgraduate education did not provide them with adequate training to support breastfeeding mothers (12).

Given the health, developmental, social, and economic advantages that breastfeeding confers to mothers, children, and society in general, breastfeeding is a critical public health initiative (3). As such, there is a need to develop, implement, and evaluate a breastfeeding education intervention for medical residents. To date, we have established a 22-member team that includes breastfeeding researchers, breastfeeding experts, postgraduate medical education leaders, medical education experts, medical residents, and breastfeeding mothers to develop, implement, and evaluate a national breastfeeding educational intervention for our medical residents in Canada.

## Methods

Our development process is informed by Kern’s systematic and iterative approach to curriculum development (13). In the following sections, we provide an overview of our process and progress to date.

### Needs assessment surveys

To establish the need for a national educational intervention, we conducted a needs assessment survey of both practicing physicians and medical residents in Canada to assess their breastfeeding knowledge, beliefs, and attitudes, as well as comfort and confidence in supporting breastfeeding mothers (12).

### Identification of learning outcomes

The 22-member team described above was selected by our investigative team. Through consultations with this team, we identified possible learning outcomes that medical residents should be able to attain after completing a breastfeeding educational intervention. To develop these outcomes, all team members were electronically asked the following open-ended question: ‘Upon completion of this resident breastfeeding educational intervention, residents should be able to …’. Two medical education experts conducted a content analysis of the responses to identify common learning outcomes. Non-responders received two electronic reminders. There was no limit on the number of learning outcomes each respondent could identify.

### Team meeting to discuss implementation and evaluation

An in-person, 2-day team meeting was held in Ottawa, Ontario, Canada on April 23 and 24, 2014. The following topics were discussed: 1) current breastfeeding education activities in various postgraduate programs; 2) results of our abovementioned needs assessment surveys; 3) possible learning outcomes for our educational intervention; and 4) possible strategies for implementing and evaluating a national educational intervention. To ensure that all team members were satisfied with our progress to date, an exit questionnaire was also completed at the end of this meeting.

## Results

### Needs assessment surveys

Our needs assessment survey of practicing physicians showed that their breastfeeding knowledge, confidence, beliefs, and attitudes are suboptimal, and that most physicians feel that their postgraduate education had not adequately prepared them to support breastfeeding mothers (12). Similar results were obtained from the needs assessment survey of the residents (results not yet published).

### Identification of learning outcomes

Upon review of the learning outcome suggestions of the team, we identified the following five potential learning outcomes for a future educational intervention:

*Upon completion of this resident breastfeeding educational intervention, residents should be able to:*

1. Assess breastfeeding technique.
2. Assist mothers in achieving proper breastfeeding technique.
3. Explain common health problems that mothers experience during breastfeeding.
4. Summarize the benefits of breastfeeding for children and mothers.
5. Mobilize appropriate resources for breastfeeding mothers.

### Team meeting to discuss implementation and evaluation

Twenty-two individuals attended the in-person, 2-day expert panel meeting. The abovementioned learning outcomes were discussed in detail. Through various facilitated small- and large-group discussions, the learning outcomes were revised and agreed upon by all meeting attendees to comprise the following:

*Upon completion of this resident breastfeeding educational intervention, residents should be able to:*

1. Assess and promote breastfeeding in all infants and children, following the evidence-based policies of the World Health Organization (WHO) Ten Steps to Successful Breastfeeding (14).
2. Recognize common child and maternal health presentations experienced during breastfeeding and contraindications to breastfeeding.
3. Support, promote, and advocate breastfeeding through various presentations of common maternal and child health issues.
4. Communicate benefits of breastfeeding for mother and child.
5. Mobilize appropriate resources for breastfeeding mothers.

Potential tools for assessing residents’ attainment of these learning outcomes were discussed, including multiple choice questions (MCQ), standardized questionnaires, objective structured clinical examinations (OSCE), as well as chart reviews and audits to assess the documentation of breastfeeding discussions and reasons for artificial milk supplementation. All meeting attendees agreed that the use of a variety of assessment tools would be preferable to allow for feedback from multiple stakeholders on the performance of residents. Learning activities were also a topic of discussion; interactive, hands-on learning and the design of learning activities targeted to the specific needs of the trainees were identified as priorities. The importance of a flexible, sustainable, and feasible intervention was emphasized to facilitate widespread implementation. Finally, mechanisms to assess the impact of the educational intervention on patients were discussed. Suggestions included surveying patients to assess their satisfaction with breastfeeding support, performing chart audits to determine appropriateness of indications for supplementation, tracking formula supplementation rates, as well as exclusive and partial breastfeeding rates. The exit questionnaire that the team members completed at the end of this meeting was overwhelmingly positive, with all agreeing that progress to date with regard to the development of this educational intervention was acceptable.

## Conclusions

To date, this systematic process has provided an opportunity to plan the initial development, implementation, and evaluation of a national breastfeeding educational intervention for medical residents. It has provided an opportunity to foster collaboration among breastfeeding researchers, breastfeeding experts, postgraduate education leaders, medical education experts, medical residents, and breastfeeding knowledge users. By allowing for the sharing of ideas and experiences, we anticipate that this future educational intervention will enhance research capabilities and create a collaborative network of various experts. The outcomes of this project will be relevant to all residents and physicians involved in the care of infants and their mothers and may impact exclusive breastfeeding rates and duration, ultimately leading to improved health outcomes in infants and their mothers.
